# Transcriptomic analysis reveals cell apoptotic signature modified by heparanase in melanoma cells

**DOI:** 10.1111/jcmm.14349

**Published:** 2019-05-02

**Authors:** Tianyi Song, Dorothe Spillmann

**Affiliations:** ^1^ Department of Medical Biochemistry and Microbiology University of Uppsala Uppsala Sweden

**Keywords:** apoptosis, cancer, gene expression, heparanase, melanoma cells

## Abstract

Heparanase has been implicated in many pathological conditions, especially inflammation and cancer, attributed to its degradation of heparan sulfate, a crucial component maintaining the integrity of the extracellular matrix. By silencing the heparanase gene (HPSE) in MDA‐MB‐435s melanoma cells, we investigated the impact of this protein on gene transcription. Transcriptome sequencing yielded a list of 279 differentially expressed genes, of which 140 were up‐regulated and 239 down‐regulated. The 140 up‐regulated genes were classified into a substantial set of gene ontology defined functions, for example, positive regulation of cell death, apoptotic process, response to cytokine, while 239 down‐regulated genes classify only into the two categories: nucleosome and nucleosome assembly. Our focus was drawn to an array of 28 pro‐apoptotic genes regulated by heparanase: real‐time PCR experiments further validated up‐regulation of EGR1, TXNIP, AXL, CYR61, LIMS2 and TNFRSF12A by at least 1.5‐fold, among which EGR1, CYR61, and TNFRSF12A were confirmed on protein level. We demonstrated significantly increased apoptotic cells by TUNEL staining upon HPSE silencing, mediated by activation of caspase 3/PARP1 pathway. The pro‐apoptotic gene expression and observation of apoptosis were extended to another melanoma cell line, MV3 cells, thus consolidating the anti‐apoptosis effect of heparanase in melanoma cells.

## 1 INTRODUCTION

Heparanase is the only endo‐β‐D‐glucuronidase that cleaves glucosiduronic bonds between glucuronate/iduronate and glucosamine residues of heparan sulfate (HS) chains resulting in release of oligosaccharides with shortened HS chains remaining on proteoglycans (PGs) on the cellsurface and in the extracellular matrix (ECM).^1^ Heparanase is synthesized and secreted as a 65 kDa pro‐heparanase that binds to HSPGs on the cell surface and is subsequently taken into the early endosome where it is processed to active heparanase by cathepsin L.^2^ Except its presence in the cytoplasm, heparanase has been reported to translocate into the cell nucleus and play an important role as part of an active chromatin complex.^3^

A compelling number of studies have tied heparanase with inflammatory diseases and cancer. Its role in inflammation is well accepted to be mediated by its remodelling of HS thereby modulating the sequestration of cytokines in the extracellular space, affecting leukocytes interaction with endothelium^4^, ^5^ and ECM and initiation of innate immune responses through interactions with toll‐like receptor 4.^6^ HPSE expression is enhanced in almost all malignant tumours examined including various carcinomas, sarcomas and haematological malignancies.^7^, ^8^, ^9^, ^10^, ^11^ Heparanase regulates pleiotropic biological activities that promote tumour growth, angiogenesis and metastasis. Numerous clinical studies have consistently demonstrated that up‐regulation of HPSE expression correlates with increased tumour size, tumour angiogenesis, enhanced metastasis and poor prognosis.^12^, ^13^, ^14^ In contrast, silencing of HPSE or treatments of tumour‐bearing mice with heparanase inhibiting compounds, markedly attenuate tumour progression.^15^, ^16^, ^17^, ^18^ However, the mechanisms underlying these effects remain to be further investigated.

Although there is a great number of publications with focus ranging from cell signalling to clinical studies of heparanase, to date there is no report of gene expression profile correlated to altered HPSE expression. Using melanoma cells that express a high level of endogenous heparanase, we demonstrates that heparanase regulates a number of genes involved in a substantial set of biological functions. Focusing on its regulation on cell apoptosis, we were able to validate the up‐regulation of pro‐apoptotic genes and display apoptosis in cell culture after silencing HPSE.

## 2 MATERIALS AND METHODS

### 2.1 Materials

ON‐TARGETplus Human HPSE siRNA‐smartpool (set of 4), ON‐TARGETplus non‐target pool siRNA, DharmaFECT 1 transfection reagent 1 were all purchased from Dharmacon (Little Chalfont, United Kingdom). Silencer Select HPSE siRNA (single) was from Thermo Scientific (Massachusetts). A Click‐iT^®^ Plus TUNEL assay kit and CellEvent^TM^ Green Detection Reagent Caspase 3/7 staining kit were from Life Technologies (California). iScript cDNA synthesis kit and SsoFast EvaGreen Supermix were purchased from BioRad (California). RNeasy Plus Mini Kit and Qproteome Nuclear Protein Kit were from QIAGEN (Hilden, Germany). High‐Capacity cDNA Reverse Transcription Kit was obtained from Applied Biosystems (California). Heparanase antibody #1453 was a gift from Israel Vlodavsky (Rappaport Faculty of Medicine, Haifa, Israel). Antibodies against Sp1 (1C6) and Histone 1 (G1) were from Santa Cruze. Anti‐caspase 3 (#9662), cleaved caspase 3 (#9661), PARP 1 (#9542) were from Cell Signalling Technology. Anti‐EGR1 (AF2818), CYR61 (MAB4055), TNFRSF12 (MAB1199) were from R&D Systems (Abingdon, UK), and anti‐GAPDH (AM4300) from Ambion. SuperSignal Duro Substrate from Thermo Scientific (Massachusetts).

### 2.2 Cell culture

T47D cells were from ATCC, MDA‐MB‐435s cells were from ATCC, and recently identified as melanoma origin.^19^ Melanoma MV3 cells (from Gefan, Shanghai, China) were originally from ATCC. The cells were further confirmed by authentication testing preceding this study (Eurofins, Germany). All cells were cultured in DMEM supplemented with 10% foetal bovine serum (FBS) in 5% CO_2_ at 37°C. Medium was changed twice a week.

### 2.3 SiRNA silencing of HPSE gene

Melanoma cells were seeded at a concentration of 1 × 10^5^/mL and maintained in complete medium for 24 hours. SiRNA silencing was achieved by the addition of Dharmafect 1 transfection reagent with an optimized smartpool of HPSE siRNAs, Silencer Select HPSE siRNA, or a control siRNA at a concentration of 30 nmol/L. After 24 hours culture media were refreshed, silencing of HPSE expression was confirmed by real‐time PCR and Western blot after 48 hours.

### 2.4 RNA preparation and sequencing

Total RNA from control siRNA and smartpool HPSE siRNA transfected cells was extracted using a QIAGEN RNA mini kit. The total RNA had a standard concentration of 200 μg/μL and a RNA‐integrity (RIN) of ≥7.0 as measured on an Agilent 2100 Bioanalyzer. Samples were sent to the National Genomics Infrastructure platform, Sweden, for easy gene‐level transcriptome sequencing by Ion AmpliSeq^TM^ technology. R version 3.3.3 was used to analyse the gene expression data. Gene ontology (GO) analysis was conducted using PANTHER, an online gene analysis tool, on which gene overrepresentation was performed for binomial test, with the Bonferroni correction for multiple testing. GO terms that met a corrected *P* ≤ 0.05 were defined as significantly enriched GO term.^20^, ^21^

### 2.5 Real‐time PCR

Total RNA was isolated from the cells by following the manufacturer's instructions. RNA (1 μg) was reversely transcribed to cDNA with a High‐Capacity cDNA Reverse Transcription Kit and diluted to a final volume of 200 μL. A 20 μL reaction mixture containing 2 μL of cDNA template, 0.5 μmol/L primers and 10 μL SsoFast EvaGreen Supermix was added to a 96‐well white‐clear plate. RT‐PCR was performed using a CFX384^TM^ RT‐PCR system with the BioRad CFX manager software version 3.0. Conditions for amplification were 95°C for 30 seconds and 40 cycles of 95°C for 5 seconds followed by 56°C for 5 seconds. The fold change of mRNA was evaluated by the relative copy number and expression ratios of targeted genes normalized to the expression of the reference gene (18S rDNA). Ratios were calculated by the relative quantification method using the CFX manager software with the equation RCN = 2−ΔΔC_t_, where ΔC_t_ = C_t_ target − C_t_ reference, and ΔΔC_t_ = ΔC_t_ test sample − ΔC_t_ control sample. The sequences of primers used are described in File S3.

### 2.6 Western blot analysis

Cells were lysed with RIPA buffer (50 mmol/L Tris, pH 7.5, 150 mmol/L NaCl, 1% Nonidet P‐40, 0.5% sodium deoxycholate, 0.1% SDS, 1 mmol/L EDTA) for 30 minutes on ice. For heparanase location analysis, the cells were fractionated with Qproteome Nuclear Protein Kit following the manufacturer's instructions. The concentration of all lysate samples was determined by BCA assay. After denaturation of proteins, samples (30 μg of total protein) were subjected to a 10% reducing gel and separated by SDS‐PAGE before transfer to polyvinylidene fluoride (PVDF) membranes by semidry blotting. Membranes were then blocked with 5% milk in TBS‐T (20 mmol/L Tris, pH 7.4, 150 mmol/L NaCl, 0.1% Tween‐20) and probed with rabbit polyclonal antibodies against heparanase, Sp1, Histone 1, caspase 3, cleaved caspase 3, PARP 1, EGR1, CYR61, TNFRSF12A or GAPDH. Signals in the blots were developed with SuperSignal Duro Substrate according to the manufacturer's instructions. Densitometry was performed using ImageLab software.

### 2.7 Apoptotic cell detection

Melanoma cells (1 × 10^5^) were plated on coverslips in 12‐well plates. For TUNEL assay, cells were fixed in 4% PFA followed by permeabilization with 0.25% Titan^®^ X‐100 for 10 min at room temperature. Fixed cells were subjected to TUNEL assay using the Click‐iT^®^ Plus TUNEL System following the manufacture's instruction. For detection of activated caspase 3/7‐positive cells, CellEvent^TM^ Green Detection Reagent was added to the medium, and cells incubated for 30 minutes at 37°C in a humidified incubator. Thereafter, cells were fixed with 4% PFA and counterstained with DAPI before imaging by fluorescence microscopy.

### 2.8 Statistics

Statistics for experimental data are expressed as mean ± standard deviation (SD) of three independent experiments. Unpaired Student's *t* test was used for statistical analysis. A *p* ≤ 0.05 was considered statistically significant.

## 3 RESULTS

### 3.1 Subcellular location of heparanase and silencing of HPSE expression in melanoma cells

**Figure 1:**
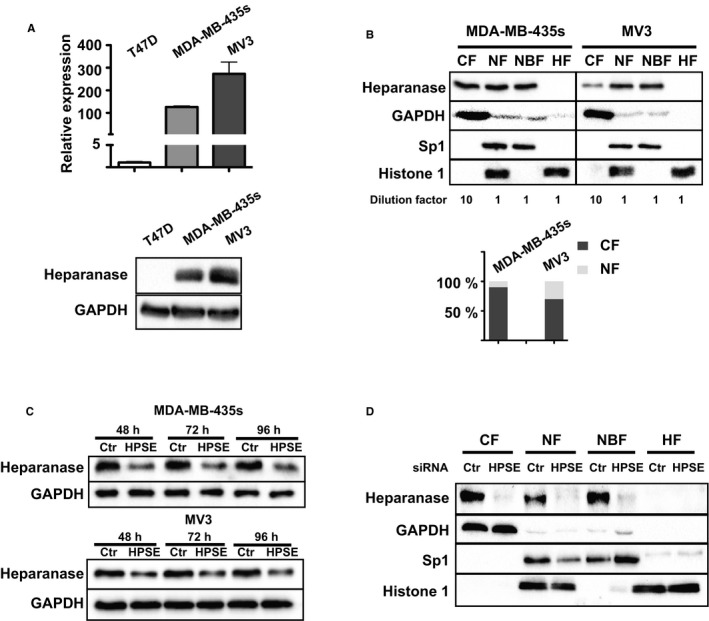
Characterization of subcellular localization of heparanase and silencing of heparanase gene (HPSE) gene in melanoma cells. (A) Real‐time PCR analysis of HPSE expression and protein detection by Western blot in T47D cells, MDA‐MB‐435s cells and MV3 cells. Relative expression of HPSE to 18S rDNA was calculated and the expression in T47D cells was set as 1. (B) MDA‐MB‐435s cells and MV3 were lysed and fractionated as described in Materials and Methods and blotted with antibodies against heparanase, cytosolic marker GAPDH, nuclear marker Sp1 and histone‐fraction marker Histone 1. CF, Cytosolic fraction; HF, Histone fraction; NBP, Nuclear binding proteins; NF, Nuclear fraction. The lysis buffer volumes for CF, NF, NBP were 500 uL, 50 uL, 50 uL and dilution factors correspondingly indicated. Relative protein amount were quantified by ImageLab and adjusted by dilution factor. Total amount of cytosolic and nuclear heparanase is set as 100%. n = 3, mean values are presented. (C) Western blot analysis of melanoma cells transfected with control siRNA (siRNA‐Ctr) or a smartpool of HPSE siRNAs (siRNA‐HPSE) after 48 h, 72 h and 96 h. (D) siRNA‐transfected MDA‐MB‐435s cells were lysed after 72 h and fractionated as indicated in (B)

The HPSE promoter in normal cells and tissues is constitutively silenced by methylation^22^, ^23^, ^24^, ^25^ and the action of p53^26^ except in placenta, activated immune cells and keratinocytes, in which heparanase is constitutively active. However, HPSE expression can be induced in a number of inflammation related pathological processes by mediators such as tumour necrosis factor (TNF) or interleukin 1β (IL‐1β).^27^, ^28^, ^29^ In this study, we screened breast cancer T47D cells, melanoma MDA‐MB‐435s cells and MV3 cells for heparanase expression. We found that melanoma MDA‐MB‐435s cells and MV3 cells expressed a remarkably high level of endogenous heparanase (Figure 1A).

To characterize the subcellular location of heparanase in the melanoma cells, we performed cell fractionation to separate cell lysates into cytosolic, nuclear and nuclear binding protein fractions. By Western blot analysis, we found the majority (roughly 90%) of heparanase to be present in the cytoplasm and detected the presence of approximately 10% of heparanase in the nucleus in MDA‐MB‐435s cells, whereas 30% of heparanase was detected in MV3 nuclei (Figure 1B). For both cell lines we were able to extract the nuclear binding proteins and identified nuclear heparanase protein mostly located in the nuclear binding protein fraction. Taking advantage of the smartpool of siRNAs targeting HPSE mRNA, we were able to eliminate heparanase to a substantial degree in both MDA‐MB‐435s and MV3 cells after 48, 72 and 96 hours confirmed on protein level by Western blot analysis (Figure 1C). Furthermore, subcellular fractionation analysis further confirmed elimination of heparanase in both cytoplasmic and nuclear compartments by silencing HPSE expression in MDA‐MB‐435s cells as shown in Figure 1D.

### 3.2 Transcriptomic analysis identifies differentially expressed genes

**Figure 2:**
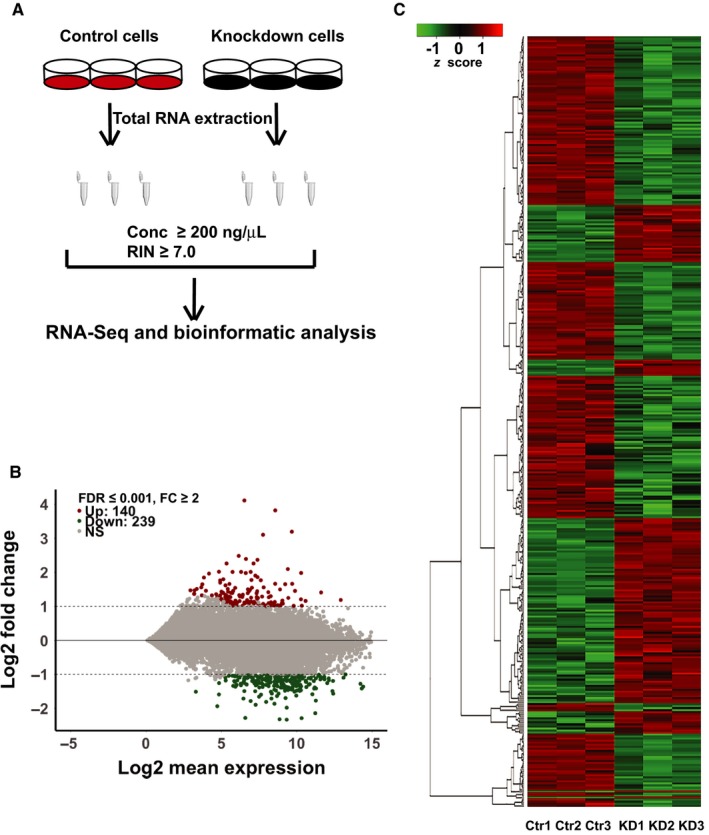
Comparative transcriptome of melanoma cells transfected with control siRNA and heparanase gene (HPSE) siRNA. (A) Description of the workflow of RNA sequencing and analysis. (B) MA‐plot of gene expression in control and HPSE siRNA‐transfected cells. Each gene is marked as an individual dot, of which 140 are up‐regulated (red) in the HPSE‐silenced cells and 239 (green) down‐regulated. Grey dots indicate genes that are not significantly differentially expressed between the two groups. The false discovery rate (FDR) is set as ≤0.001 and fold‐change (FC) threshold as 2. (C) Heat map of 379 differentially expressed genes (|log2 FC| ≥1, FDR ≤ 0.001, n = 3). Red colour intensity indicates up‐regulation, and green colour down‐regulation. Dendrogram clustering on the *y*‐axis indicates genes with similar expression profiles. Colour key indicates gene count and expression level. Control siRNA transfected (Ctr) and HPSE siRNA transfected cells (KD)

For RNA‐sequencing, triplicate RNA samples from cells transfected with control siRNA and HPSE siRNA were prepared. cDNA libraries were constructed following the workflow shown in Figure 2A. After the removal of adaptor sequences, ambiguous nucleotides, and low quality sequences, a total of 92 166 793 raw reads were generated for further analysis with the mean read length of 115 and 116 bp, respectively. Clean reads from the six samples were aligned to the hg19 AmpliSeq Transcriptome ERCC version 1.0 with *homo sapiens* reference genes to generate count based gene expression values. The mapping rate to the reference genome ranged from 95.09% to 95.91%.

To identify the differentially expressed genes that were associated with HPSE expression, all sequenced genes were screened between the cells transfected with control siRNA and HPSE siRNA to remove genes with low counts by defining false discovery rate (FDR) > 0. By doing this, 13 644 genes were identified as a reference gene list for functional enrichment analysis. We used a criterion that marks genes for which the fold change (FC) of HPSE silenced over control silenced is ≥2, FDR ≤0.001, as up‐regulated, and those for which the ratio is ≤0.5, FDR ≤ 0.001, as down‐regulated. Applied to the data from six samples, this yielded a list of 279 differentially expressed genes, of which 140 were up‐regulated and 239 down‐regulated, presented in an MA‐plot (Figure 2B; File S1).

To visualize gene expression data, the expression raw counts were log2 transformed corrected by library size, and the differentially expressed genes (|log2 FC| ≥ 1, FDR ≤ 0.001, n = 3) were extracted and displayed as a heat map (Figure 2C). Based on the similarity of their gene expression patterns, the heat map clusters genes that show biological signatures associated to the regulation by heparanase.

### 3.3 Functional analysis of differentially expressed genes reveals up‐regulation of an array of pro‐apoptotic genes

**Figure 3:**
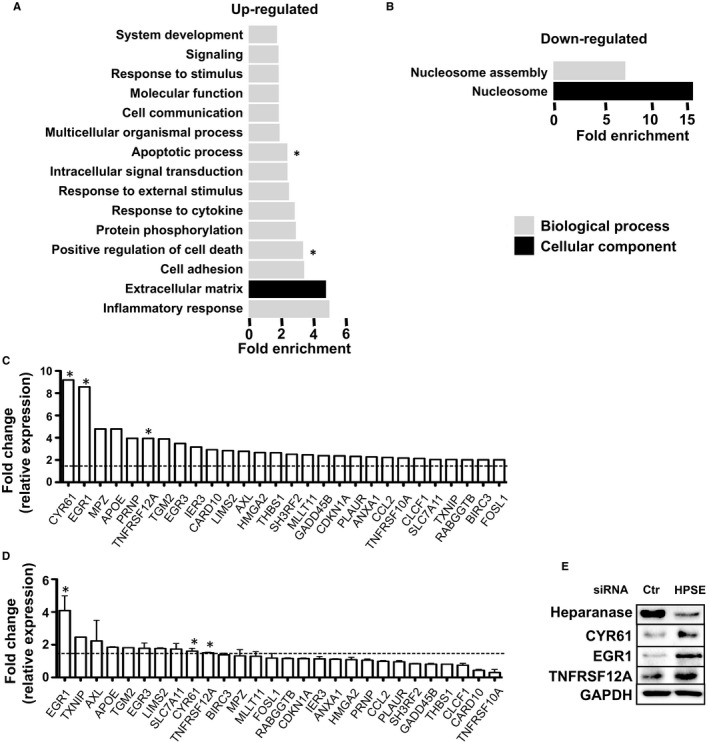
Gene ontology classification of differentially expressed genes regulated by silencing of heparanase gene (HPSE) expression. (A) Gene ontology (GO) analysis of up‐ and (B) down‐regulated genes after silencing of HPSE in MDA‐MB‐435s cells by terms of biological process and cellular component. *X*‐axis **i**ndicates functional fold enrichment calculated by binomial test,* P* < 0.01. (C) Listing of an array of 28 pro‐apoptotic genes classified by GO term positive regulation of cell death and apoptotic process. *Y*‐axis indicates fold change comparing HPSE silenced cells with control cells. Dashed line indicates 1.5‐fold change. (D) Validation of expression of the 28 pro‐apoptotic genes by real‐time PCR. n = 3 biological repeats, * indicates the selected genes for further validation by Western blots. Dashed line indicates 1.5‐fold change. (E) Validation of up‐regulation of selected genes including CYR61, EGR1 and TNFRSF12A on protein level by Western blots. N = 3 biological repeats, representative blots are shown

To identify the potential functions of the differentially expressed genes, we used the online gene analysis tool PANTHER to perform a gene ontology (GO) term analysis on the list of the differentially expressed genes. These genes were classified into the categories: molecular function, biological process, and cellular component^21^. The functional enrichments of up‐ and down‐regulated genes are presented separately (Figure 3A,B). In addition, we listed the GO classification and the complete GO enrichment analysis of differentially expressed genes in the File S2. Genes were annotated in GO terms using the terminology provided by PANTHER, primarily inflammatory response, extracellular matrix, cell adhesion, positive regulation of cell death for up‐regulated genes, and nucleosome and nucleosome assembly for down‐regulated genes. To our surprise, we found a substantial set of genes that were up‐regulated in HPSE silenced cells, which suggests that heparanase could act as a negative regulator of transcription; those genes classify in the GO term of positive regulation of cell death, apoptotic process, response to cytokine, response to external stimuli, response to stimuli (Figure 3A).

Many studies have detailed the involvements of heparanase in acute and chronic inflammation by modification of the extracellular matrix or direct regulation of inflammatory cell function.^30^ As expected, genes related to inflammatory response were the most enriched among all significant GO terms. Notably, heparanase exhibited a strong impact on the expression of genes involved in positive regulation of cell death and apoptotic process, suggesting a potential biological relevance (Figure 3A). By zooming into the specific genes from the interesting terms, our attention was drawn onto an array of 28 pro‐apoptotic genes including extracellular matrix proteins such as LIM zinc finger domain containing 2 (LIMS2), cysteine‐rich angiogenic inducer 61 (CYR61), AXL receptor tyrosine kinase (AXL), transcription factors such as early growth response protein 1 (EGR1), thioredoxin interacting protein (TXNIP) and cell death receptor as tumour necrosis factor receptor superfamily 12A (TNFRSF12A) among the genes with elevated expression after elimination of heparanase (Figure 3C).

To verify the pro‐apoptotic genes regulated by heparanase, we performed real‐time PCR on the 28 genes comparing HPSE silenced cells using smartpool siRNAs to control cells. The results validated that among other genes the expression of EGR1, CYR61 and TNFRSF12A was consistently up‐regulated in HPSE silencing cells as shown in Figure 3D. In parallel, Western blot analysis further confirmed the up‐regulation of those genes on protein level as shown in Figure 3E.

### 3.4 Silencing of HPSE expression in melanoma cells induces caspase 3/PARP1‐mediated apoptosis

Heparanase was shown to promote tumour cell proliferation, migration and evasion of apoptosis. Earlier studies have shown that cells with high levels of heparanase have enhanced Akt, STAT, p38, Erk and EGF receptor signalling activity, which may provide survival signals to the cells.^31^, ^32^, ^33^ To elucidate the biological relevance of the array of pro‐apoptotic genes revealed by RNA sequencing, we transfected MDA‐MB‐435s cells with control or smartpool HPSE siRNAs and performed TUNEL staining of the cells after 48, 72 and 96 hours. TUNEL staining demonstrated that cells with HPSE silencing showed significantly increased numbers of apoptotic cells, with a dramatic amount of cell apoptosis after 96 hours.

**Figure 4:**
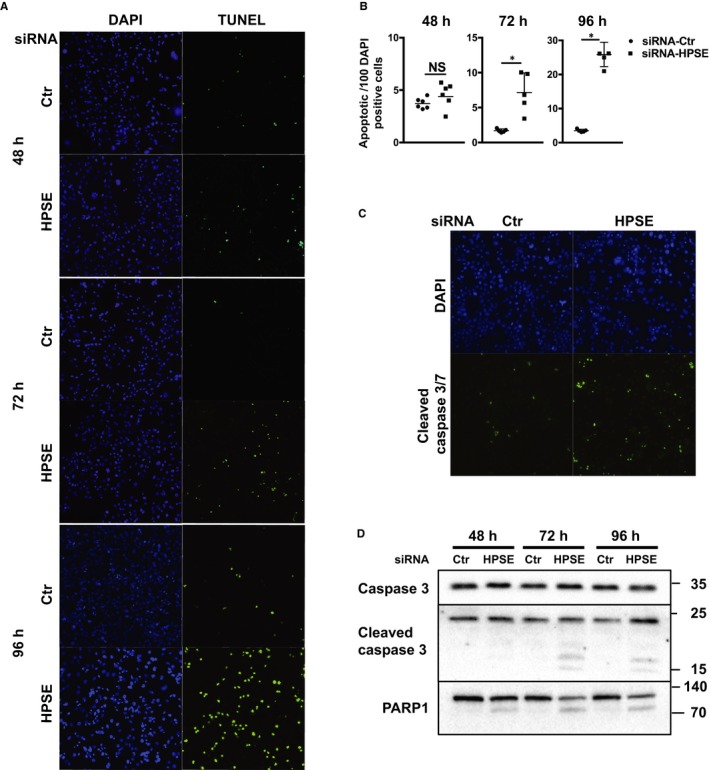
Silencing of heparanase gene (HPSE) expression in MDA‐MB‐435s cells induces caspase 3/PARP1 mediated apoptosis. (A) TUNEL staining (green) of MDA‐MB‐435s cells 48, 72 and 96 h after transfection with control or HPSE siRNA, and counterstained by DAPI (blue). (B) TUNEL‐staining positive cells were quantified manually and normalized to number of DAPI positive cells quantified by ImageJ. Quantification is presented as apoptotic cells per 100 DAPI positive cells from 3 biological repeats, *P < 0.01. (C) Detection of cleaved caspase 3/7 by fluorescent antibody of control and HPSE silenced cells after 72 h as described in Methods. Cells were counterstained with DAPI. (D) Western blots of whole cell lysates for apoptotic executer cleaved caspase 3 and downstream PARP1 as indicated after transfecting cells with control or HPSE siRNA for 48, 72 and 96 h. Representative blots are shown from three independent experiments

A study done using xenografted pancreatic cancer cells revealed that heparanase inhibitor PG545 significantly increased apoptosis via cleaved caspase 3, along with decreased cell proliferation, reduced microvessel density, disrupted vascular function, and elevated intratumoural hypoxia.^16^ To consolidate our finding of increased apoptosis, the cells were subjected to fluorescent staining for cleaved caspase 3/7 after 72 hours of gene silencing. Increased staining of cleaved caspase 3/7 was exhibited in HPSE silenced cells, compared to control cells (Figure 4C). Furthermore, Western blot analysis of the whole cell lysates using antibodies against caspase 3, cleaved caspase 3 and PARP1, revealed fragmentation of caspase 3 and PARP1 (Figure 4D) occurring in HPSE silenced cells from day 2, but nearly undetectable in control cells, suggesting involvement of caspase 3 in the apoptosis induced by elimination of heparanase. The apoptosis of MDA‐MB‐435s cells was carefully consolidated by silencing HSPE expression using a different single siRNA (Figure S1A,B), as well as involving the activation of caspase 3/PARP1 pathway (Figure S1C). A similar pattern of up‐regulation of EGR1, CYR61 and TNFRSF12A was concomitantly observed on protein level shown by Western blots (Figure S1D).

To extend the apoptotic phenotype observed in MDA‐MB‐435s cells, another melanoma cell line, MV3 cells were included and evaluated in this study. Apoptotic cells detected by TUNEL staining were consistently induced following 72 hours gene silencing targeting HPSE using both single siRNA and smartpool of siRNAs (Figure S2A,B), via activation of caspase 3/PARP1 (Figure S2C). In order to provide potential mechanistic insight through linkage of differential expression of pro‐apoptotic genes to melanoma cell apoptosis, we examined the expression of 28 pro‐apoptotic genes in MV3 cells after silencing HPSE using smartpool of siRNAs. By real‐time PCR, we were able to replicate the up‐regulation of EGR1, TXNIP, AXL, CYR61, LIMS2 and TNFRSF12A as indicated in MDA‐MB‐435s cells (Figure S2D). Accordingly, increased protein levels of EGR1, CYR61 and TNFRSF12A were also observed following HPSE gene silencing in MV3 cells shown by Western blots (Figure S2E).

## 4 DISCUSSION

Except binding to a plethora of cytokines including the members of the fibroblast growth factor family, transforming growth factors, bone morphogenetic proteins and interleukins, HS also interacts with ECM molecules like collagens, laminin, fibronectin, to contribute to the structural integrity of the ECM, assists in preservation of proper tissue organization and inhibits cellular invasion by promoting cell–cell and cell–ECM interactions.^34^, ^35^ HS cleavage by heparanase results thus in liberation of bioactive molecules, modulation of the cellular microenvironment and disassembly of the ECM, including basement membranes underlying epithelial and endothelial cells, leading to structural modification that loosens ECM barriers and enables cell dissemination.^36^, ^37^

However, the function of heparanase is not limited to the extracellular surroundings, which has been studied extensively. Heparanase can also interfere with gene transcription directly by binding to nuclear DNA^38^ or indirectly by controlling histone H3 methylation patterns.^39^, ^40^ The overexpression of heparanase in melanoma cell lines prompted us to study its role on cancer cells per se.^38^, ^41^ Accordingly, melanoma MDA‐MB‐435s cells and MV3 cells expressed remarkably high levels of endogenous heparanase (Figure 1A). In both cell lines, there was easily detectable heparanase located in the nucleus, of which the majority was associated with the fraction of nuclear binding proteins (Figure 1B). Using siRNAs targeting HPSE expression, we could eliminate a considerable level of heparanase after 48 hours from the cells, as well as from the nuclear compartment (Figure 1C,D).

Ion AmpliSeq^TM^ technology provided us a useful tool to shed light on the transcriptional profile of HPSE silenced cells compared to control cells. To our surprise, we identified only 140 genes that were up‐regulated in HPSE silenced cells (Figure 2B), but classified into a substantial set of the GO term functions including positive regulation of cell death, apoptotic process, response to cytokine, response to external stimuli, response to stimuli (Figure 3A), which suggests that heparanase may act as a negative regulator of those biological processes. In contrast, 239 genes that were down‐regulated fall into the only two categories of nucleosome and nucleosome assembly (Figures 2B and 3B).

Acquired resistance toward apoptosis is a hallmark of most and perhaps all types of cancer.^42^ Heparanase promotes cancer cell survival, tumour growth and chemoresistance. Accordingly, siRNA mediated silencing of HPSE expression in melanoma MDA‐MB‐435s cells led to significant increase in apoptosis (Figure 4A,B), which was mediated by activation of caspase 3/PARP1 pathway (Figure 4C,D). This observation was further extended to MV3 cells, where a similar pattern of apoptosis was induced, albeit to a lesser extent (Figure S2A,B), with the involvement of caspase 3/PARP1 pathway as well (Figure S2C).

Recently, the ability of heparanase to drive exosome secretion and alter exosome composition, has been linked to tumour progression. 43 And the presence of heparanase in autophagosomes confers the cells more resistance to stress and chemotherapy associated with increased autophagy.^44^ Furthermore, heparanase was able to cooperate with Ras to enhance number and size of induced breast cancer lesions, suggesting pro‐tumorigenic properties.^45^ We have suggested that heparanase may modulate tumour cell apoptosis via direct interference with apoptotic pathways, thus altering the susceptibility to apoptosis‐inducing factors possibly during carcinogenesis and anti‐cancer therapy, although the apoptosis‐modulating role of heparanase has not been studied sufficiently.

In this study, siRNA mediated silencing of HPSE expression in melanoma cells induced significantly increased expression of an array of genes classified as GO terms of positive regulation of cell death and apoptotic process (Figure 3A). Carefully screening of those differentially up‐regulated genes resulted in a listing of 28 pro‐apoptotic genes, for example, EGR1, TXNIP, AXL, CYR61, LIMS2 and TNFRSF12A with at least twofold increased expression in MDA‐MB‐435s cells (Figure 3C). Furthermore, real‐time PCR experiments on the 28 genes comparing HPSE silenced cells with control cells validated among other genes the up‐regulated expression of EGR1, TXNIP, AXL, CYR61, LIMS2 and TNFRSF12A by at least 1.5‐fold in HPSE silenced cells (Figure 3D), and for EGR1, CYR61 and TNFRSF12A also confirmed on protein level (Figure 3E). Of note, a similar effect was previously observed upon adding heparanase to rat cardiomyocytes, where adding exogenous latent heparanase was reported to down‐regulate pro‐apoptotic genes such as TNF superfamily 10 and its receptor TNFRSF10B. By acting directly on the apoptotic receptor and ligand, heparanase was able to provide protection of the cells against high glucose and H_2_O_2_ induced cell‐death.^46^

The elucidation of transcription regulation by heparanase revealed its biological implication in apoptosis, and an array of pro‐apoptotic genes modified by heparanase. It should be noted that although some canonical pathways and factors that trigger apoptosis have already been identified, the specific mechanisms that particular cells use to cooperate or circumvent the apoptotic pathways remain to be elucidated, presumably reflecting the diversity of apoptosis—inducing signals that cancer cells populations encounter during their evolution to a malignant state. Nevertheless, we were able to obtain reproducible up‐regulation of genes including EGR1, TXNIP, AXL, CYR61, LIMS2 and TNFRSF12A in MDA‐MB‐435s and MV3 melanoma cell lines and significant increase in protein levels as well (Figure S2D,E), suggesting potential implication in other melanoma cells. Although the apoptosis evading mechanisms of different cancer cells may vary regarding the factors that come into play, heparanase's play in conferring a common apoptosis resistant fate may extend to other cancer cells.

In summary, our study provides for the first time a gene expression profile associated with altered HPSE expression. Among the multiple biological processes, HPSE silencing revealed up‐regulation of an array of pro‐apoptotic genes and established an anti‐apoptosis effect of heparanase involving caspase 3/PARP1 activation, supporting its role for promoting survival, inducing chemo‐resistance. Taken together, our results add new possibilities to so far probably underscored role and unclear mode of action of heparanase in modifying gene transcription, and potentially oncogenic properties previously reported.^45^

## DATA SHARING

Data are available on request from the authors.

## CONFLICT OF INTEREST

The authors declare that they have no competing interests.

## AUTHORS’ CONTRIBUTIONS

Tianyi Song designed the research study, performed the experiments, analysed the data and wrote the manuscript; Dorothe Spillmann supervised the project and contributed with discussions, revised the manuscript.
