# Supplementary material for: Transcriptomic analysis reveals cell apoptotic signature modified by heparanase in melanoma cells
Source: J Cell Mol Med. 2019 May 2;23(7):4559–68. doi: 10.1111/jcmm.14349 (PMC6584584; doi:10.1111/jcmm.14349)
Supplement: Supplementary file 1 [file JCMM-23-4559-s001.pdf]

Suppl. File 1

Up-regulated genes				Down-regulated genes					
AKAP9	GPR157	PLAUR	WFDC3	ASIC1	CUEDC1	HIST1H2BH	MEF2A	RGS10	UNG
ALDH1L1	HMGA2	PLEKHA8P1	ZNF114	ACY1	CUX1	HIST1H2BM	MFHAS1	RNPEPL1	VAMP3
ANXA1	HSPA4L	PLSCR4	ZNF165	ADCY6	DAG1	HIST1H2BN	MGAT4B	RPS6KA1	VEPH1
ANXA3	IER3	PLXNA3	REXO1L2P	ADCY9	DCP2	HIST1H3C	MMP8	RRBP1	VOPP1
APLN	IFRD1	PMEL	RPL23AP64	ADD1	DCT	HIST1H3F	MOB3B	SART1	ZNF25
APOE	IGFBP6	PPIL4		AGBL5	DDHD1	HIST1H3I	MXD3	SCARNA10	ZNF618
APOL3	IKBKG	PPP1R1B		AK5	DDHD2	HIST1H3J	MYO19	SCARNA18	ZNF704
ARHGAP29	IL8	PRNP		ALG6	DEPTOR	HIST1H4B	NACC1	SCARNA3	ZRANB1
AXL	KCNQ1OT1	PRSS16		AMMECR1	DOM3Z	HIST1H4D	NCEH1	SCARNA5	ACSS1
B3GALT2	KLC3	RAB11FIP4		ARHGAP5	DUSP16	HIST1H4I	NDUFAF3	SDC1	ANKRD36BP1
BIRC3	KRT14	RAB17		ASF1B	E2F2	HIST2H2AB	NFATC2	SEC14L2	LOC100129034
BRSK1	KRT9	RABGGTB		ATP6V1E2	E2F4	HIST2H2AC	NLRX1	SEMA5A	NUDT3
DNAAF3	KYNU	RASA4		ATP8B2	EHD4	HIST2H2BE	NME4	SESN3	POMGNT1
CARD10	LIF	RBPMS2		BAALC	ELF4	HPSE	NOV	SGCD	TUG1
CCDC103	LIMS2	RNF182		BMF	ENTPD6	HSDL1	NRAS	SLC16A13	
CCDC69	LINC00326	S100A16		BNIP3L	FABP7	HTRA2	NRBP2	SLC24A5	
CCL2	LIPH	S100A2		VWA9	FADS2	HYOU1	P2RX7	SLC25A39	
CD22	LOC440028	SCAND3		C1orf85	FAM160B1	ID3	PACSIN3	SLC35F1	
CDKN1A	LOC646329	SCARF2		C21orf58	FAM195B	IL12RB2	PDK1	SLC9A8	
CLCF1	LOC646471	SESN2		C2orf29	FAM3C	IL6R	PELI3	SNORA23	
CLSTN3	KRTAP2-3	SGMS2		C5orf15	FBXL7	INSIG1	PGD	SNORA38	
COL1A2	LUM	SH2D3A		SMIM3	FGF14	IRS2	PHPT1	SNX18	
CTGF	MAP1B	SH3RF2		RABL6	FHDC1	ITPK1	PIGT	SOX2	
CTSS	MAP4K5	SLC7A11		CABLES2	FKBP8	KANK2	PKI55	SREBF1	
CXCL16	MAPK6	SMPDL3B		CADM1	FST	KCNAB1	PLA2G15	STK32A	
CYP1A1	MAPRE2	SNAPC1		CALD1	FTH1	KIAA1644	PLCD1	STRN4	
CYP4F11	METTL7A	SNCG		CAMK2N2	FYN	KLF9	PLEKHF2	TCEAL2	
CYR61	MLLT11	SYBU		CAMKK2	GET4	KLHL36	PLEKHH1	TEF	
DDAH1	MMP25	SYT1		CAPZA1	GLDC	LCP2	POM121	TFDP1	
DDX58	MMP3	TAF5		CCM2	GLT25D2	LGALS3BP	POM121C	TMEM133	
DLG4	MOG	TGM2		CCRL1	GNA12	LIMD1	PPIL1	TMEM47	
DNAJC12	MPZ	THBS1		CD99L2	GPNMB	LIMK1	PPIP5K2	TMTC1	
EGR1	MRGPRX4	TINAGL1		CDC25B	GSTA4	LOC100132077	PPP4C	TNC	
EGR3	MYEOV	TM4SF1		CDK2AP2	GTF3C2	LOC100506474	PQLC1	TNFRSF19	
EPHA2	MYH15	TM4SF18		CEP68	HDAC4	LOC643837	PRDM16	TNFRSF21	
FAM18B1	NAT8L	TM4SF19		CERK	HERC3	LPCAT2	PRIMA1	TP53INP2	
FAM8A1	NAV3	TMEM106A		CHAF1A	HIRA	LRP2	PRMT2	TPCN2	
FOS	NEK9	TNFRSF10A		CKAP4	HIST1H1B	LRRC1	PRPS2	TRAPPC1	
FOSL1	NEXN	TNFRSF12A		CNOT6	HIST1H1C	LRRC23	PRR14	TSC22D4	
GADD45B	NT5E	TPM1		CPM	HIST1H1D	LSM14B	PTMA	TTYH3	
GBP1	NTM	TPM2		CREBL2	HIST1H2AC	LZTS1	PTTG1IP	TYR	
GBP3	PAQR7	TRIM16L		CRELD2	HIST1H2AD	MAEA	PUS1	UBE2Q1	
GEM	PDCD1LG2	TXNIP		CRK	HIST1H2AG	MAMLD1	RAD9A	UBQLN4	
GLIPR1	PDE2A	UCN2		CRTC3	HIST1H2AM	MAP2	RAP2B	UGDH	
GOLT1A	PLA2G16	VGF		CTBP1	HIST1H2BB	MBNL3	RFXANK	UGT8	
