# Supplementary material for: Transcriptomic analysis reveals cell apoptotic signature modified by heparanase in melanoma cells
Source: J Cell Mol Med. 2019 May 2;23(7):4559–68. doi: 10.1111/jcmm.14349 (PMC6584584; doi:10.1111/jcmm.14349)
Supplement: Supplementary file 2 [file JCMM-23-4559-s002.pdf]

Suppl. File 2

GO analysis of up-regulated genes

**GO biological process complete**	Number of genes	Fold Enrichment (+)	P value
inflammatory response	12	5.31	2.36E-02
defense response	21	3.13	2.55E-02
cell adhesion	20	3.65	4.46E-03
biological adhesion	20	3.62	4.89E-03
positive regulation of cell death	19	3.58	1.13E-02
regulation of cell death	32	2.58	3.36E-03
positive regulation of protein phosphorylation	21	3.11	2.74E-02
positive regulation of phosphorylation	22	3.10	1.69E-02
positive regulation of phosphate metabolic process	22	2.92	4.31E-02
positive regulation of phosphorus metabolic process	22	2.92	4.31E-02
response to cytokine	22	3.03	2.42E-02
response to organic substance	42	2.03	2.26E-02
response to chemical	54	1.84	9.97E-03
response to external stimulus	32	2.65	1.98E-03
intracellular signal transduction	31	2.56	6.55E-03
signaling	59	1.75	1.10E-02
cell communication	60	1.74	1.11E-02
regulation of apoptotic process	29	2.55	1.78E-02
regulation of programmed cell death	29	2.52	2.10E-02
regulation of multicellular organismal process	40	2.03	4.33E-02
regulation of cell communication	48	1.99	5.30E-03
regulation of molecular function	45	1.99	1.45E-02
regulation of response to stimulus	56	1.98	3.90E-04
regulation of signaling	48	1.97	7.66E-03
system development	54	1.87	5.15E-03
multicellular organism development	56	1.72	4.62E-02
multicellular organismal process	68	1.58	4.49E-02
Unclassified	6	.46	0.00E00
**GO cellular component complete**			
extracellular matrix	11	5.09	1.61E-02
extracellular region	47	1.88	6.97E-03
Unclassified	2	.24	0.00E00

GO analysis of down-regulated genes

**GO biological process complete**	Number of genes	Fold Enrichment	P value
nucleosome assembly	12	7.41	8.57E-04
chromatin assembly	12	6.63	2.77E-03
DNA packaging	12	5.15	3.69E-02
chromatin assembly or disassembly	12	5.61	1.56E-02
nucleosome organization	13	6.22	1.84E-03
Unclassified	20	.95	0.00E00
**GO cellular component complete**			
nucleosome	14	14.36	2.78E-09
DNA packaging complex	14	12.57	1.60E-08
protein-DNA complex	14	5.79	2.65E-04
chromatin	21	2.75	4.12E-02
Unclassified	11	.82	0.00E00
