# Supplementary material for: Transcriptomic analysis reveals cell apoptotic signature modified by heparanase in melanoma cells
Source: J Cell Mol Med. 2019 May 2;23(7):4559–68. doi: 10.1111/jcmm.14349 (PMC6584584; doi:10.1111/jcmm.14349)
Supplement: Supplementary file 3 [file JCMM-23-4559-s003.pdf]

Gene (human)	sense 5’-3’	antisense 5’-3’
CYR61	CTCGCCTTAGTCGTCACCC	CGCCGAAGTTGCATTCCAG
MPZ	CATCGTGGTTTACACCGACAG	TGGAAGATCGAAATGGCATCTCT
Egr1	GGTCAGTGGCCTAGTGAGC	GTGCCGCTGAGTAAATGGGA
ApoE	GTTGCTGGTCACATTCCTGG	GCAGGTAATCCCAAAAGCGAC
PRNP	CGAGCTTCTCCTCTCCTCA	ACAAAGAGAACCAGCATCCA
TNFRSF12A	CTGGCTCCAGAACAGAAAGG	GGGCCTAGTGTCAAGTCTGC
TGM2	GAGGAGCTGGTCTTAGAGAGG	CGGTCACGACACTGAAGGTG
Egr3	GACATCGGTCTGACCAACGAG	GGCGAACTTTCCCAAGTAGGT
IER3	CAGCCGCAGGGTTCTCTAC	GATCTGGCAGAAGACGATGGT
CARD10	CCTCATCCTCTGACAGCGTG	CCAGACACCCGAATAGCCAG
LIMS2	GCACCGGCACTATGAGAAGAA	ACGGGCTTCATGTCGAACTC
AXL	ATCAGCTTCGGCTAGGCAG	TCCGCGTAGCACTAATGTTCT
HMGA2	GACGTCGGGCATTCATATAGG	TTGGTGTTCTAAACAGAGGATTCACT
THBS1	AGACTCCGCATCGCAAAGG	TCACCACGTTGTTGTCAAGGG
SH3RF2	GGACGCCTGTGTTTTCCAAC	TGAGCGCACTCCATCCAGA
MLLT11	GGGACCCTGTGAGTAGCCAG	CTGCAGTTGCTTGCCCGA
GADD45B	GGGAAGGTTTTGGGCTCTCT	CGGTCACCGTCCGCATCTT
CDKN1A	TGTCCGTCAGAACCCATGC	AAAGTCGAAGTTCCATCGCTC
PLAUR	TGTAAGACCAACGGGGATTGC	AGCCAGTCCGATAGCTCAGG
ANXA1	GCGGTGAGCCCCTATCCTA	TGATGGTTGCTTCATCCACAC
CCL2	TTTCAACGAGCCAGACTTCAAC	GAGGCCACGCAAGTAACACA
TNFRSF10A	GCGGGGAGGATTGAACCAC	CGACGACAAACTTGAAGGTCTT
CLCF1	TTTCAACGAGCCAGACTTCAAC	GAGGCCACGCAAGTAACACA
SLC7A11	ATGCAGTGGCAGTGACCTTT	GGCAACAAAGATCGGAACTG
TXNIP	GGTCTTTAACGACCCTGAAAAGG	ACACGAGTAACTTCACACACCT
RABGGTB	CGGAGAAGTTACCAGATGTATGC	TACGCAGTTTCTCTCTATCAATCC
BIRC3	AAGCTACCTCTCAGCCTACTTT	CCACTGTTTTCTGTACCCGGA
FOSL1	CAGGCGGAGACTGACAAACTG	TCCTTCCGGGATTTTGCAGAT
