# Supplementary material for: Transcriptomic analysis reveals cell apoptotic signature modified by heparanase in melanoma cells
Source: J Cell Mol Med. 2019 May 2;23(7):4559–68. doi: 10.1111/jcmm.14349 (PMC6584584; doi:10.1111/jcmm.14349)
Supplement: Supplementary file 4 [file JCMM-23-4559-s004.pdf]

Supplementary information to

Transcriptomic analysis reveals cell apoptotic signature modified by heparanase in melanoma cells

Tianyi Song[1], Dorothe Spillmann[1]

[1]Department of Medical Biochemistry and Microbiology, University of Uppsala, Uppsala, Sweden

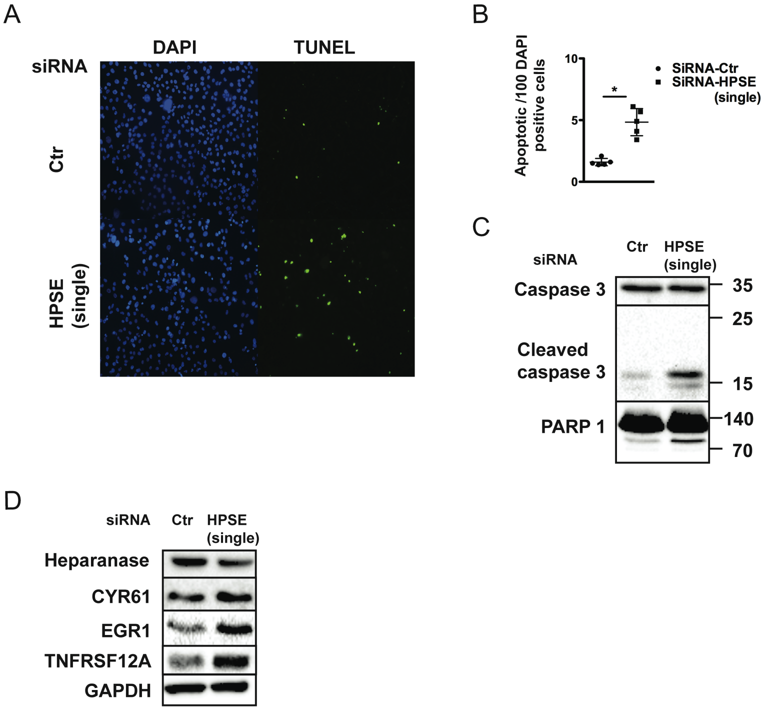

Suppl.Figure 1: Confirmation of apoptosis and pro-apoptotic gene expression induced by silencing HPSE expression in MDA-MB-435s cells. A) TUNEL staining (green) of MDA-MB-435s cells 72 h after transfection with control or single HPSE siRNA and counterstained by DAPI (blue). Ctr: control siRNA, HPSE (single): single HPSE siRNA. B) TUNEL-staining positive cells were quantified manually and normalized to number of DAPI positive cells quantified by ImageJ. Quantification is presented as apoptotic cells per 100 DAPI positive cells from 3 biological repeats, * $p < 0.01$. C) Western blots of whole cell lysates for apoptotic executer cleaved caspase 3 and downstream Parp 1 as indicated. Representative blots are shown from 3 independent experiments. D) Validation of HPSE silencing and up-regulation of selected interest genes including CYR61, EGR1 and TNFRSF12A on protein level by western blots. n=3 biological repeats, representative blots are shown.

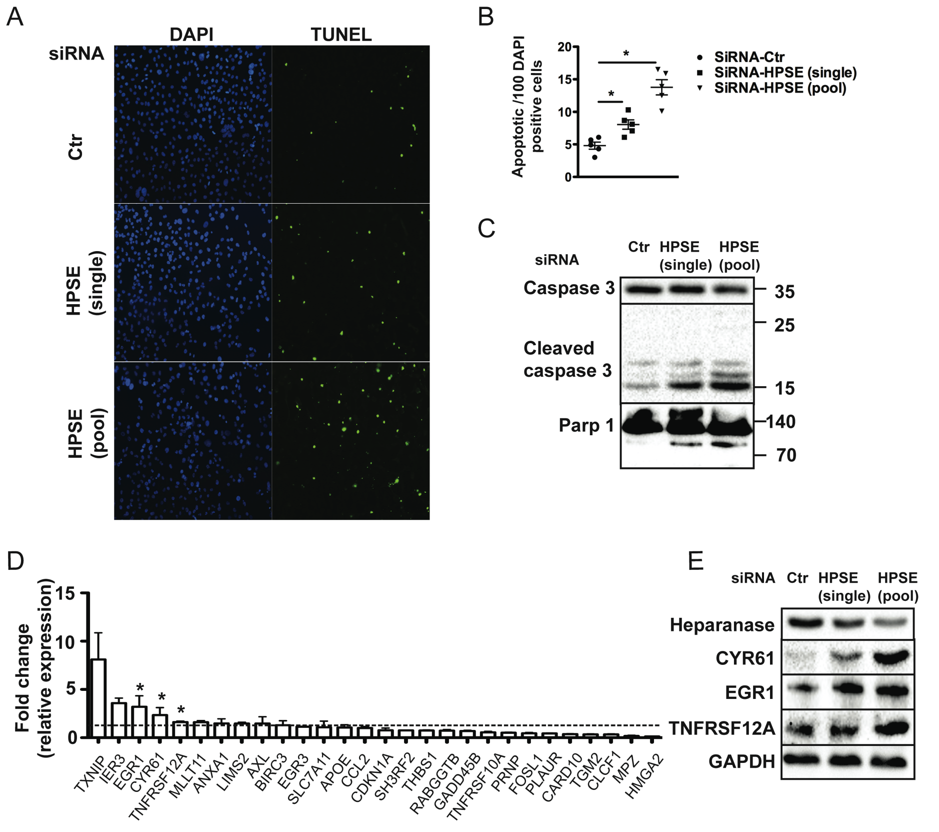

Suppl.Figure 2: Validation of apoptosis and pro-apoptotic gene expression induced by silencing HPSE expression in MV3 cells.
A) TUNEL staining (green) of MV3 cells 72 h after transfection with control, single HPSE siRNA or smartpool HPSE siRNAs and counterstained by DAPI (blue). Ctr: control siRNA, HPSE (single): single HPSE siRNA. HPSE (pool): smartpool HPSE siRNAs. B) TUNEL-staining positive cells were quantified manually and normalized by number of DAPI positive cells quantified by ImageJ. Quantification is presented as apoptotic cells per 100 DAPI positive cells from 3 biological repeats, * $p < 0.01$.
C) Western blots of whole cell lysates for apoptotic executer cleaved caspase 3 and downstream PARP 1 as indicated. D) Validation of gene expression of the 28 pro-apoptotic genes by real-time PCR. Y axis indicates fold change comparing HPSE silenced cells using smartpool HPSE siRNAs with control cells. Dashed line indicates average 1.5 fold change, * indicates the selected interest genes for further validation by Western blots. E) Validation of HPSE silencing and up-regulation of selected interest genes including CYR61, EGR1 and TNFRSF12A on protein level by Western blots- N=3 biological repeats, representative blots are shown.

Suppl.File 1

List of differentially expressed genes regulated by heparanase

All raw counts of sequenced genes were screened in R, version 3.3.3. Differentially expressed genes between cells transfected with control siRNA and HPSE siRNA were identified applying the criterion of fold change (FC) of HPSE silencing over control is ≥2, FDR ≤0.001, it yielded a list of 279 differentially expressed genes, of which 140 were up-regulated and 239 down-regulated.

Suppl.File 2

Functional analysis of differentially expressed genes
An online gene analysis tool PANTHER was used to perform a gene ontology (GO term analysis on the list of the differentially expressed genes. These genes were classified into the categories: molecular function, biological process, and cellular component. The functional enrichments of up-regulated and down-regulated genes are analyzed and presented separately as marked.

Suppl.File 3
Sequences of primers used for real-time PCR.
